# Linking Alpha-Synuclein to the Actin Cytoskeleton: Consequences to Neuronal Function

**DOI:** 10.3389/fcell.2020.00787

**Published:** 2020-08-12

**Authors:** Marina I. Oliveira da Silva, Márcia A. Liz

**Affiliations:** ^1^Instituto de Ciências Biomédicas Abel Salazar (ICBAS), Universidade do Porto, Porto, Portugal; ^2^Neurodegeneration Group, Instituto de Biologia Molecular e Celular (IBMC) and Nerve Regeneration Group, Instituto de Investigação e Inovação em Saúde (i3S), Universidade do Porto, Porto, Portugal

**Keywords:** alpha-Synuclein, Parkinson’s disease, actin cytoskeleton, actin-binding proteins, cofilin-1

## Abstract

Alpha-Synuclein (αSyn), a protein highly enriched in neurons where it preferentially localizes at the pre-synapse, has been in the spotlight because its intraneuronal aggregation is a central phenomenon in Parkinson’s disease. However, the consequences of αSyn accumulation to neuronal function are not fully understood. Considering the crucial role of actin on synaptic function and the fact that dysregulation of this cytoskeleton component is emerging in neurodegenerative disorders, the impact of αSyn on actin is a critical point to be addressed. In this review we explore the link between αSyn and actin and its significance for physiology and pathology. We discuss the relevance of αSyn-actin interaction for synaptic function and highlight the actin-depolymerizing protein cofilin-1 as a key player on αSyn-induced actin dysfunction in Parkinson’s disease.

## Introduction

In the last years, a large body of evidences point to the neuronal cytoskeleton damage as a major contributor to neurodegeneration (Eira et al., 2016). From the various cytoskeleton dysfunctions, alterations in microtubule (MT) stability are a main causative agent in several neurodegenerative disorders and include: (i) variations in the levels of tubulin post-translational modifications with a consequent impact on axonal transport (Dompierre et al., 2007; d’Ydewalle et al., 2011; Zhang et al., 2014; Qu et al., 2017; Magiera et al., 2018), and (ii) dysregulation of MT associated proteins, such as the key example of tau hyperphosphorylation. This modification promotes tau detachment from MTs, what either impacts on axonal transport (Alonso et al., 1997) or leads to recruitment of MT severing enzymes causing axon degeneration (Qiang et al., 2006). Concerning the actin cytoskeleton, dysregulation of this component causing neurodegeneration mostly derives from actin accumulations as the case of cofilin-actin rods (Minamide et al., 2000; Munsie and Truant, 2012).

In the case of Parkinson’s disease (PD), the most common synucleinopathy, characterized by the intraneuronal accumulation of aggregated αSyn, several reports demonstrated αSyn-induced alterations of the microtubule cytoskeleton dynamics and axonal transport defects (Carnwath et al., 2018; Prots et al., 2018). As αSyn is a protein that impacts on the synapse both physiologically and pathologically, an association between the protein and the actin cytoskeleton, a cell component crucial for synaptic function, has been suggested. This review will focus on the current knowledge on the interaction between αSyn and actin and actin-binding proteins (ABPs) concluding with a critical perspective of the implication of these interactions in health and disease. Addressing this topic will certainly contribute with new insights into αSyn-related pathology opening new lines of research targeting neurodegeneration.

## Alpha-Synuclein: From Physiology to Pathology

αSyn was originally identified in the *Torpedo* electric organ and is one of the most abundant proteins in the brain (Maroteaux and Scheller, 1991). It belongs to the synuclein family of proteins, a group of small soluble proteins that transiently bind to neuronal membranes, which also includes β-Synuclein and γ-Synuclein (Clayton and George, 1998). αSyn is a 140 amino-acid protein containing three distinct motifs (Maroteaux et al., 1988): the N-terminal contains seven repeats of a 11-residue sequence (XKTKEGVXXXX) responsible for αSyn interaction with vesicles containing phospholipids, the central region contains a non-amyloid component (NAC) sequence which is relatively hydrophobic and prone to aggregation, and the intrinsically unstructured C-terminus region, responsible for multiple protein interactions (Emamzadeh, 2016). αSyn is a natively unfolded protein implying that it lacks a secondary organized structure (Weinreb et al., 1996), what enables the protein to adopt several conformations comprising an unstructured soluble cytosolic form, an α-helical membrane bound structure or a β-sheet-like prone to aggregate conformation (Burre et al., 2013).

αSyn is ubiquitously expressed but highly enriched in the nervous systems (Lavedan, 1998). In neurons, αSyn is enriched in the pre-synaptic terminals where it interacts with synaptic vesicles and participates in several steps of the vesicle cycle comprising trafficking, docking, fusion, and recycling after exocytosis, therefore playing a central role in the regulation of the synaptic transmission (Scott and Roy, 2012; Diao et al., 2013; Wang et al., 2014). Notably, synuclein proteins were proposed to have a major impact in the long-term function of synaptic transmission, as αβγ-Syn triple-knockout mice showed reduced SNARE (SNAP REceptor)-complex assembly, and presented neuropathological signs and shortened lifespan (Burre et al., 2010). Interestingly, although αSyn plays an important role at the pre-synapse, it is one of the last proteins to be targeted to the synapse during development, suggesting that its physiologic function is not focused on the synapse formation but more directed to its maintenance (Cheng et al., 2011). Although αSyn role at the synapse is the most well studied physiological function, a number of additional cellular localizations have been described, including mitochondria, nucleus, endoplasmic reticulum, Golgi complex, and cytoskeleton components, suggesting that the protein contributes to the regulation of innumerous cellular processes (Burre et al., 2018).

αSyn is a protein deeply associated with disease since it was described as the main component of Lewy Bodies (LBs), the pathological hallmark of synucleinopathies (Spillantini et al., 1997), a group of diseases caused by αSyn aggregation and pathology from which PD is the most common one. Although being a cytosolic protein, and its intra-neuronal accumulation resulting in neurodegeneration, it is now known that cell-to-cell transmission of αSyn aggregates also occurs contributing to the progression and propagation of the disease (Karpowicz et al., 2019). Further supporting αSyn link with pathology, familial forms of PD are related with duplication, triplications and point mutations (mainly A30P and A53T mutations) in the *SNCA* gene, encoding for αSyn, which increase the aggregation potential of the protein (Burre et al., 2018).

αSyn aggregated species were shown to induce neurotoxicity through several processes: (i) affecting membrane permeabilization of several cell components, including plasma membrane and endoplasmic reticulum (Colla et al., 2012), mitochondria (Parihar et al., 2009), and vesicle membranes (Volles and Lansbury, 2002; Burre et al., 2018); (ii) increasing reactive oxygen species (ROS) production (Parihar et al., 2009) and Ca^2+^ influx (Danzer et al., 2007); and (iii) disrupting protein synthesis machinery and degradation systems, namely the autophagy-lysosomal and the ubiquitin-proteasomal systems (Lindersson et al., 2004; Garcia-Esparcia et al., 2015). αSyn accumulation was also shown to negatively affect SNARE-complex assembly and disassembly impairing neurotransmitter release and leading to decreased neuronal excitability and synaptic firing, what culminates in synaptotoxicity (Vekrellis et al., 2011). In the same line, αSyn overexpression in neurons decreased spine density and impaired spine dynamics (Blumenstock et al., 2017). Interestingly, synaptic structure and function is highly dependent on actin dynamics, suggesting an impact of αSyn on that cytoskeleton component.

## Actin Cytoskeleton: a Critical Component for Neuronal Function

The integrity of the cytoskeleton is crucial for neuronal maintenance and function and depends on a critical regulation of its components: actin filaments, microtubules and intermediate filaments. The actin cytoskeleton, the component of interest in this review, is composed of actin filaments (F-actin) that are formed by the association of globular actin (G-actin) to a growing polymer (Mitchison and Cramer, 1996). Actin monomers polymerize/depolymerize from the actin filament constituting the fundamental process driving actin dynamics (Carlier, 1998). There is a considerable number of ABPs regulating actin dynamics among which are: (i) nucleation factors (formins and Arp2/3) that promote the assembly of G-actin into filaments and the development of branched networks (Bugyi et al., 2006; Korobova and Svitkina, 2008); (ii) actin-monomer binding proteins (profilin) that provide new subunits to the filament enhancing the assembly of G-actin into F-actin (Mockrin and Korn, 1980); (iii) proteins that bundle (fascin) or crosslink (α-actinins and filamins) the actin filaments (Tseng et al., 2004); (iv) tropomyosins which are able to regulate actin dynamics through binding along F-actin and interaction with other ABPs or by direct contact of F-actin with different tropomyosin isoforms (Gunning et al., 2008; Schevzov et al., 2012); (v) spectrins that are localized at subcortical regions linking actin cytoskeletal meshwork to membrane receptors (Burridge et al., 1982); (vi) capping proteins (adducin) which bind to actin filaments blocking their growth (Matsuoka et al., 2000) and (vii) severing proteins (actin depolymerizing factor (ADF)/cofilin-1 and gelsolin) which control the rate of actin polymerization (Weeds et al., 1991; Andrianantoandro and Pollard, 2006).

In neurons, actin acquires several structural rearrangements that are differentially distributed in the cell. Lamellipodia is an actin structure characterized by a branched network of short actin filaments while filopodia is composed of long parallel bundles of actin filaments (Neukirchen and Bradke, 2011). Lamellipodia and filopodia are actin arrangements enriched in the more dynamic neuronal structures including the growth cone, dendrites and dendritic spines. In the growth cones, besides filopodia and lamellipodia, there is, in the transition zone, an actomyosin contractile structure named actin arcs which are perpendicular to F-actin and are suggested to interact with microtubules and allow them to invade the growth cone (Schaefer et al., 2002). In dendritic spines, actin is the core structure providing the architecture for the formation, stability, motility, and morphology of the spines (Basu and Lamprecht, 2018). Actin patches are structurally similar to lamellipodia and are found in the axons and dendrites (Korobova and Svitkina, 2010; Spillane et al., 2011). It is suggested that they promote locally actin filopodia formation, as these actin patches are not motile structures (Spillane et al., 2011). More recently, a component of the neuronal subcortical cytoskeleton was described, the actin rings (Xu et al., 2013). This structure is along the axon and dendrites and is composed by a ring of short actin filaments capped by adducin and distanced by ∼190 nm by spectrin (Xu et al., 2013). More recently, non-muscle myosin II was proposed to regulate the axonal actin ring contraction and expansion (Costa et al., 2020). While actin rings are considered to give mechanical support to neurons, there is a dynamic pool of actin in the structure of the axon, termed actin trails, which have been suggested to be composed of actin “hotspots” and provide for a flexible actin cytoskeleton network in the axon (Ganguly et al., 2015).

In mature neurons a dysregulation of the actin cytoskeleton has tremendous implications for spine density, morphology and function (Zhang and Benson, 2001). An emergent number of studies have also reported the abundance and relevance of actin and ABPs in the pre-synaptic terminals, where actin dynamics plays essential functions on the vesicle pool organization, synaptic vesicle mobilization and exocytosis and posterior endocytosis (Rust and Maritzen, 2015). The crucial roles of actin at the synapse turns it an important protein to study in the context of neurodegenerative disorders where synaptic dysfunction is a central causing agent.

## αSyn-Actin Cytoskeleton Link: Impact on Neuronal Health and Disease

### Evidences of αSyn Interaction With Actin and Actin-Binding Proteins

The most αSyn recognized physiological function is on the synaptic vesicle cycle, a process where actin plays a key role. This functional “proximity” between αSyn and actin boosted the research on identifying an interaction between the two proteins. This interaction was investigated mainly in *in vitro* assays, either with cell-free or with cell-line approaches, which although were critical for the demonstration of the interaction of αSyn with actin and actin-binding proteins (Zhou et al., 2004; Peng et al., 2005; Esposito et al., 2007; Sousa et al., 2009; Welander et al., 2011; Lee et al., 2012), lack the validation in primary neuronal cultures which would be a more relevant scenario. In this respect, in a cellular PD model using rotenone-treated dopaminergic neurons, it was observed an increase in the expression levels of F-actin and αSyn. However, a putative interaction between the two proteins was not explored in that context (Mattii et al., 2019). Further supporting an interaction, αSyn and actin co-immunoprecipitated in rat brain homogenates under physiological conditions (Sousa et al., 2009).

Additionally, proteomic studies demonstrated alterations on the expression levels of several ABPs in models of αSyn overexpression in *D. melanogaster* (Xun et al., 2007a,b) and *C. elegans* (Ichibangase et al., 2008). Moreover, gelsolin was found in LBs from PD and Dementia with Lewy Bodies (DLB) patients and was shown to have a positive effect on αSyn aggregation in the presence of high Ca^2+^ concentrations (Welander et al., 2011).

### αSyn Impact on Actin Dynamics: Cofilin-1 Involvement

Based on the data demonstrating a putative interaction between αSyn and actin, it was investigated whether αSyn directly binds to actin modulating actin dynamics. *In vitro* cell-free assays showed that WT αSyn decreased the rate of polymerized actin, an effect suggested to occur due to the αSyn-mediated sequestration of the actin monomers. Interestingly, this effect was decreased in the presence of high concentrations of Ca^2+^, a scenario mimicking a stimulated state of neurons. In opposite, A30P aSyn, increased actin polymerization and stabilization of the actin filaments (Sousa et al., 2009). Validation of the impact of αSyn on actin dynamics was performed in studies with neuronal cell lines and primary cultures of hippocampal neurons, expressing either WT or A30P αSyn, which demonstrated that physiologically WT αSyn regulates actin dynamics, while the pathologic A30P aSyn disrupts the actin cytoskeleton (Sousa et al., 2009).

The proposed physiologic impact of WT αSyn on actin dynamics, and the fact that both αSyn and actin play a role on the synapse, raise the question of whether αSyn-actin interaction might regulate neurotransmitter homeostasis. Supporting this hypothesis, a report demonstrated a critical dependence of the interaction of αSyn with the actin cytoskeleton for the trafficking and transport activity of norepineprhine transporters (Jeannotte and Sidhu, 2008).

Concerning the pathological impact of αSyn on the actin cytoskeleton, further studies showed that the extracellular addition of high concentrations of WT or A30P αSyn to hippocampal neurons induced a stabilization of the actin cytoskeleton, by increasing the number of lamellipodia and filopodia and resistance to depolymerization, with the mutant protein having a more pronounced effect. These αSyn-induced actin alterations were mediated by the activation of the actin signaling pathway Rac1/PAK2/LIMK/cofilin-1 and to require GRP78, an endoplasmic reticulum chaperone present at the cell membrane of several cell types (Bellani et al., 2014). Cofilin-1 is an actin depolymerizing protein which activity is negatively regulated by phosphorylation in the Serine 3 residue, promoted by several kinases, including LIMK (Arber et al., 1998). As such, pathologic αSyn induced cofilin-1 phosphorylation leading to inactivation of its depolymerizing action and consequently to actin stabilization. Importantly, this outcome was recapitulated using fibroblasts from PD patients carrying multiplications of the *SNCA* gene (Bellani et al., 2014), which presented a threefold increase in the phospho-cofilin 1/cofilin 1 ratio and an increased number and thickness of actin stress fibers structures (Bellani et al., 2014).

Additional work with primary hippocampal neurons confirmed a negative effect of pathologic αSyn on actin dynamics, mediated by cofilin-1 inactivation, which impacted on neuronal functions such as axon elongation and migration (Tilve et al., 2015). Additionally, and also supporting αSyn-induced cofilin-1 inactivation, in a glaucoma animal model, consisting on elevated intraocular pressure which results in retinal neurodegeneration, the intravitreal injection of αSyn antibodies hampered neurodegeneration, an effect that was suggested to involve upregulation of cofilin-1 (Teister et al., 2017).

The presented studies claim that pathologic concentrations of αSyn induce cofilin-1 inactivation resulting on actin stabilization and neuronal dysfunction. However, cofilin-1 was also placed in the context of αSyn-induced neurodegeneration in a different scenario. In a study addressing the mechanisms of protein aggregates entry in cells, downregulation of cofilin-1 decreased αSyn aggregates entry, while both, the silencing of ROCK1 and the pharmacological inhibition of Rho, increased aggregate entry (Zhong et al., 2018). These observations suggested Rho-ROCK1-LIMK-Cofilin-1 pathway as a relevant signaling cascade triggering αSyn aggregates entry in the host cells (Zhong et al., 2018). Regardless of the context, it is remarkable the impact of αSyn in the actin cytoskeleton through cofilin-1.

### αSyn-Induced Disruption of the Actin Cytoskeleton in *in vivo* Models of PD

In the previous sections the analysis of the effect of αSyn on the actin cytoskeleton was mainly derived from cell-based studies. It is important to understand what happens *in vivo* by using disease models. In this respect, a report using a PD *Drosophila* model based on the neuronal overexpression of αSyn, validated the pathologic impact of the protein on the actin cytoskeleton, as αSyn transgenic flies showed increased F-actin and the presence of rod-shaped actin-cofilin rich inclusions in whole-mounted brains (Ordonez et al., 2018). Rod structures were also observed in the brainstem region of a PD mouse model expressing the A53T mutant form of αSyn, and in the cingulate cortex region of a DLB patient (Ordonez et al., 2018). Following experiments in fly demonstrated that the disruption of the actin cytoskeleton induced by αSyn was mediated by its interaction with α-spectrin, and resulted on the mislocalization of the mitochondrial fission protein Drp1 and subsequent mitochondrial dysfunction (Ordonez et al., 2018). This study pointed to a critical interaction between αSyn and α-spectrin resulting in actin dysfunction which consequently affects mitochondria. Additionally, and although not highly explored, the study also shows the scenario of αSyn induction of cofilin-actin rods. These are structures formed upon localized cofilin-1 activation (by dephosphorylation), leading to its association to F-actin and promoting the formation of short actin filaments saturated with cofilin-1. Rod formation has been mainly studied in the context of Alzheimer’s disease (AD) and shown to have a tremendous impact in neurons causing synaptic dysfunction, blocking axonal transport, and exacerbating mitochondrial membrane potential loss what culminates in cognitive impairment (Bamburg and Bernstein, 2016). Considering the impact of cofilin-actin rods on neurodegeneration, the pathologic relevance of rod formation upon αSyn overexpression should be further addressed. In this respect, one study reported the presence of oxidized γ-Synuclein in cofilin-actin rods in the thalamus of mice subjected to traumatic brain injury (Surgucheva et al., 2014).

## Discussion

The current revision summarizes the studies supporting the link between αSyn and the actin cytoskeleton. This is an important topic as while the interplay between αSyn and the microtubules was recently reviewed (Carnwath et al., 2018; Calogero et al., 2019), the link αSyn-actin was less explored. Considering the αSyn structural features responsible for the interaction with cytoskeleton components, it was described that αSyn is a functional microtubule-associated protein with its C-terminal region suggested to be responsible for the interaction with microtubules (Alim et al., 2004; Cartelli et al., 2016). In the case of αSyn interaction with actin, although it is implied by the available literature, no structural features underlying this interaction have been explored to date what deserves future investigation.

One important question raised by this review is whether physiologically αSyn, by regulating actin dynamics, impacts on the actin-derived functions on synaptic transmission. In this respect, while there are studies with αSyn KO mice showing decreased SNARE-complex assembly and changes in synaptic structure and size (Burre et al., 2010; Greten-Harrison et al., 2010), other reports showed no major defects in synaptic function in αSyn KO mice (Abeliovich et al., 2000; Chandra et al., 2004; Chadchankar and Yavich, 2011). Taking this into consideration, it would be important to explore neuronal actin dynamics and actin organization in synaptic structures in αSyn KO mice. This would clarify whether the αSyn-actin link acts on the physiology of the synapse, what could explain the impairment of synaptic activity in PD.

The most striking point of the present review is the highlight of cofilin-1 as a central player in αSyn-induced neuronal dysfunction. Cofilin-1 depolymerizing activity upon actin is crucial for synaptic function since the remodeling of the pre- and post-synapses intimately relies on actin dynamics (Pontrello et al., 2012). As such, we might hypothesize that the reported αSyn-activation of the actin signaling pathway Rac1/PAK2/LIMK/cofilin-1 via GRP78 (Bellani et al., 2014) which results on cofilin inactivation, and consequent blockage of actin dynamics (Sousa et al., 2009; Bellani et al., 2014) occurs at the pre-synapse, and might contribute for the synaptic dysfunction observed in the several synucleinopathies. An additional pathway by which pathogenic αSyn might cause synaptic dysfunction is *via* the induction of cofilin-actin rods formation (Cichon et al., 2012), which was observed in a PD *Drosophila* model (Ordonez et al., 2018). In AD, Aβ-induced formation of rods occurs through a pathway involving the cellular prion protein (PrP^*C*^) and NADPH oxidase (NOX) (Walsh et al., 2014), which results in the dysregulation of cofilin activity *via* oxidation and dephosphorylation, and consequent formation of cofilin-actin rods. Interestingly, αSyn was shown to interact with PrP^*C*^ to induce synaptic dysfunction (Ferreira et al., 2017). This finding raises the question of whether, similarly to what occurs in AD, αSyn-induced rod formation is mediated through a PrP^*C*^-dependent pathway culminating on disruption of synaptic activity.

While the literature suggests that αSyn might impact on neuronal function, through modulation of cofilin-1 activity, is still unclear whether αSyn induces an activation or inactivation of the ABP. Interestingly, a similar scenario was seen in AD where cofilin-1 activity was shown to be regulated in multiple ways depending on the pathogenesis context (Kang and Woo, 2019). Importantly, inhibition of cofilin activity or expression was shown to have ameliorative effects in AD (Deng et al., 2016). In the case of αSyn-induced neurodegeneration, it will be important to analyze the phosphorylation status of cofilin-1 in different pathological scenarios and cellular contexts, as well as the impact of cofilin-actin rods formation for neuronal function. These studies will be critical to point cofilin-1 as novel therapeutic target to prevent neurodegeneration in synucleinopathies.

Another point that stands out from the studies reported in this review is that the few reported experiments in primary neurons were performed with cultures of hippocampal neurons (Sousa et al., 2009; Bellani et al., 2014; Tilve et al., 2015). Although this observation suggests that additional studies should be performed with primary cultures of dopaminergic neurons, the cell type mainly affected in typical motor PD, research on the impact of αSyn on actin in hippocampal neurons is also essential considering the symptomology of dementia which has been linked to Syn pathology in the hippocampus leading to neuronal dysfunction (Hall et al., 2014).

**FIGURE 1:**
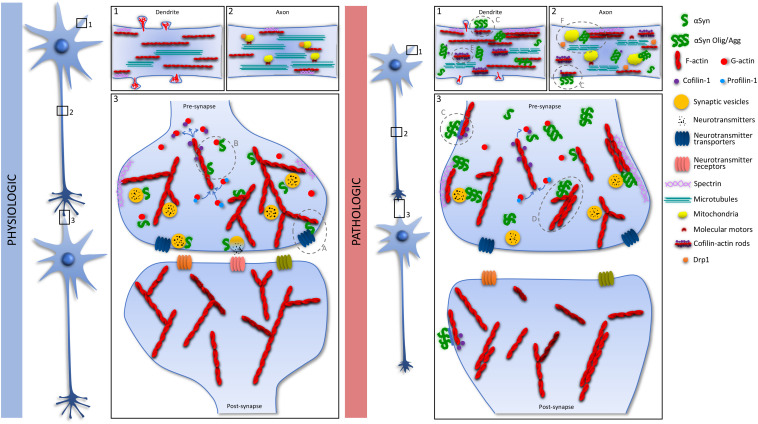
Schematic representation of the link between αSyn and actin. Under physiologic conditions αSyn is mainly concentrated at the pre-synapse and interacts with synaptic vesicles at the several stages of the vesicle cycle. At the pre-synapse αSyn-actin interaction was described as being crucial for the trafficking and transport activity of neurotransmitter transporters (Physiologic, Panel 3; **A**). Additionally, we suggest that the reported αSyn regulation of actin dynamics might contribute for the proper actin-derived functions at the pre-synapse (Physiologic, Panel 3; **B**). Under pathologic conditions, overexpression and misfolding of αSyn affects several cellular processes. At the cell membrane, extracellular misfolded αSyn uses a pathway culminating in cofilin-1 activation and actin remodeling to enter the cells (Pathologic, Panels 1–3; **C**). Intracellularly, synaptic dysfunction might occur due to the αSyn-induced stabilization of the actin cytoskeleton (Pathologic, Panel 3; **D**), through inactivation of cofilin-1 or αSyn-induced cofilin-actin rod formation (Pathologic, Panel 1; **E**), that also affects axonal transport. Additionally, αSyn interaction with spectrin (Pathologic, Panel 2; **F**), disrupts the actin cytoskeleton with consequent mislocalization of Drp1 and mitochondrial impairment.

In summary, this review presents a critical perspective in the αSyn impact on the actin cytoskeleton. The literature here revised strongly suggests that αSyn interacts/modulates actin and ABPs what has consequences to pathophysiology, as summarized in Figure 1. Nevertheless, this topic requires further investigation what might be of extremely importance in the context of both health and disease.

## Author Contributions

MO and ML conceived the structure and content and wrote the manuscript. MO produced the figure. Both authors contributed to the article and approved the submitted version.

## Conflict of Interest

The authors declare that the research was conducted in the absence of any commercial or financial relationships that could be construed as a potential conflict of interest.
